# Nonspecific Adverse Events in Knee Osteoarthritis Clinical Trials: A Systematic Review

**DOI:** 10.1371/journal.pone.0111776

**Published:** 2014-11-03

**Authors:** Yun Hyung Koog, Jin Su Lee, Hyungsun Wi

**Affiliations:** 1 Honam Research Center, Medifarm Hospital, Suncheon, Republic of Korea; 2 Department of Oriental Medicine, Medifarm Hospital, Suncheon, Republic of Korea; 3 Department of Rehabilitation, Medifarm Hospital, Suncheon, Republic of Korea; University of Michigan, United States of America

## Abstract

**Background:**

Adverse events (AEs) derived from nonspecific activity of treatments can impair the validity of trials, and even make it difficult to identify specific AEs associated with treatments. To better understand these nonspecific AEs, we investigated the AEs in placebo groups by using knee osteoarthritis clinical trials.

**Methods:**

Randomized, placebo-controlled, knee osteoarthritis trials were identified by searching electronic databases. We determined the rate of patients with AEs and the rate of dropouts caused by AEs in the active and placebo groups. Furthermore, we calculated the rate of patients for individual AEs in the placebo groups. Finally, we performed secondary analyses to identify the factors associated with these rates.

**Results:**

Overall, 272 papers reporting 281 trials were included in the analysis. The rates of patients with AEs were 31.8% in the active groups and 27.4% in the placebo groups. The rate of the placebo groups accounted for 86.2% of the rate of the active groups. The rates of dropouts caused by AEs were 5.2% in the active groups and 4.8% in the placebo groups. The rate of the placebo groups accounted for 92.3% of the rate of the active groups. AEs in the placebo groups included a number of clinical conditions, with elevated alanine aminotransferase (0.59%; 95% CI: 0.46 to 0.77) being the most common objective outcome and headache (4.48%; 95% CI: 4.20 to 4.79) being the most frequent subjective outcome. The rate of patients with AEs and the rate of dropouts caused by AEs were associated with the treatment type, delivery route, and study design.

**Conclusions:**

The nonspecific AEs substantially accounted for the development of AEs in the active groups and included conditions involving the entire body.

## Introduction

Osteoarthritis is one of the most prevalent musculoskeletal diseases affecting the elderly. Because it is characterized by the progressive degeneration of synovial joint structure [1], it causes pain, loss of motion, and physical disability, thus impairing quality of life. Current treatment strategies also aim to alleviate joint pain, reduce physical disability, and limit the progression of joint damage [2]. In line with this concept, basic efforts to establish treatment guidelines often focus on treatment efficacy [2], [3].

Nonetheless, adverse events (AEs) are an essential aspect to be considered when evaluating treatments for clinical use [4]. Although AEs arise as direct results from the specific activity of the treatments, they can also be derived from nonspecific activity. Such nonspecific AEs can often hinder patients from adhering to their treatments, thus leading to attrition bias. Furthermore, if the nonspecific AEs are high, the specific AEs of the treatments can be difficult to identify.

To better understand nonspecific AEs, numerous reviews have investigated the AEs in placebo groups, linking them to the nocebo response [5]–[15]. When participants are informed about possible AEs caused by active treatments, and thus expect them during the trial, some participants may respond negatively to placebos. It was reported that osteoarthritic patients gained health benefits with placebos, indicating that the placebo response is significant in osteoarthritis [16]. Since the nocebo and placebo responses may occur simultaneously (e.g., acupuncture [17]–[20]), osteoarthritic patients may also experience a significant nocebo response.

On the basis of these considerations, we attempted to investigate nonspecific AEs using randomized osteoarthritis trials. This study focused on knee osteoarthritis, because the number of placebo-controlled trials focusing on the knee is almost 10 times higher than those focusing on any other site [16]. Moreover, the anatomy, physiology, risk factors, and responses to the same treatments may be different for each site [21], [22].

## Methods

### Search strategy and study selection

The search strategy used in this study has been described previously [23], [24]. Briefly, the final search was performed in PubMed, SCOPUS, and the Cochrane Central Register of Controlled Trials up to December 2011, using knee osteoarthritis-related terms with the limits set to trials. For example, in PubMed, we used terms [“knee arthritis” OR “knee osteoarthritis” OR “gonarthritis” OR “gonarthrosis”], with limits to randomized controlled trials and humans. A search was also carried out in the Cochrane Review database to identify additional papers. The search was then expanded to all studies referenced in the papers. On the basis of a previous dataset of trials [23]–[28], two authors (HW and JSL) jointly selected randomized, placebo-controlled, clinical trials that investigated treatment efficacy or safety. Only trials written in English were included. We excluded trials investigating test treatments as an adjunct therapy (e.g., tramadol trial as an adjunct [29]), because nonspecific AEs may be affected by the addition of other treatments.

### Data extraction

AEs were defined as “any untoward medical occurrences that may present during treatment … but which do not necessarily have a causal relationship with this treatment” [30]. Specifically, any AEs occurring in placebo groups were defined as nonspecific AEs. First, we randomly selected 50 trials, and two authors (YHK and MG) independently extracted data, including the number of patients experiencing any AE and number of dropouts caused by AEs. After discussions, the two authors independently extracted data from the whole trials. In instances of multiple data found at more than one time point, the time point when both variables were reported together was preferred. If only one variable was reported, the time point that was considered primary was chosen. This time point was considered as trial duration. For crossover trials, only data acquired before the crossover were extracted. The inter-rater reliability was kappa = 0.905 for recording the number of patients with adverse events and kappa = 0.845 for recording the number of dropouts caused by adverse events. To determine the total number of patients in the two groups in each trial, initially randomized patients were considered. In addition, two authors independently extracted data for AEs and patient number corresponding to each AE. After referencing previous reviews [5]–[8], the final data were determined at team meetings. These data were rechecked by another author (HW).

### Data analysis

First, the rate of patients with AEs and the rate of dropouts caused by AEs in the two groups were calculated using logit transformation. The proportions were then combined using a random effects model [31]. Summary rates were calculated by back-transforming the combined proportions, and presented as percentages with 95% confidence intervals. Heterogeneity was assessed using an I^2^ test [32]. Small study effects were evaluated using a funnel plot and Egger's regression test [33].

Second, the frequencies in separate trials were summed to obtain the cumulative number of patients corresponding to each AE. If trials did not report relevant AEs, we assumed that there were no patients complaining of those AEs in the trials. Summary rates were then expressed as percentages with 95% confidence intervals. As large sample sizes were used for the calculations, the intervals were determined using the Agresti-Coull method [34]. We categorized these rates into objective and subjective rates.

### Secondary analyses

We examined whether the rates of patients with AEs and dropouts caused by AEs in the placebo groups were affected by trial qualities (i.e., allocation concealment, provider blinding, patient blinding, and attrition rate). Allocation concealment was considered adequate if the researchers responsible for patient selection could not predict the next treatment for a patient. Provider or patient blinding was considered adequate if the provider or patient could not predict the treatment for a patient. Attrition rate was considered adequate if the attrition rate of all initially randomized patients was below 15% [26].

We further observed whether the rates were influenced by the following factors: treatment type; delivery route; trial duration; flare design (yes vs. no/unclear); prohibition of usual analgesics during the trial (yes vs. no/unclear); and allowance of escape medication (yes vs. no/unclear). A meta-regression analysis with a random effects model was performed [35]. To this end, individual treatments were categorized according to the treatment type (pharmacological, non-pharmacological, and mixed) and delivery route (oral, topical, injection/puncture, and surgical). Subsequently, both factors were coded in a descending order of rates. Statistical significance was defined as values of P<0.05. STATA 11.0 software (StataCorp LP, Texas, USA) was used for all analyses.

## Results

**Figure 1:**
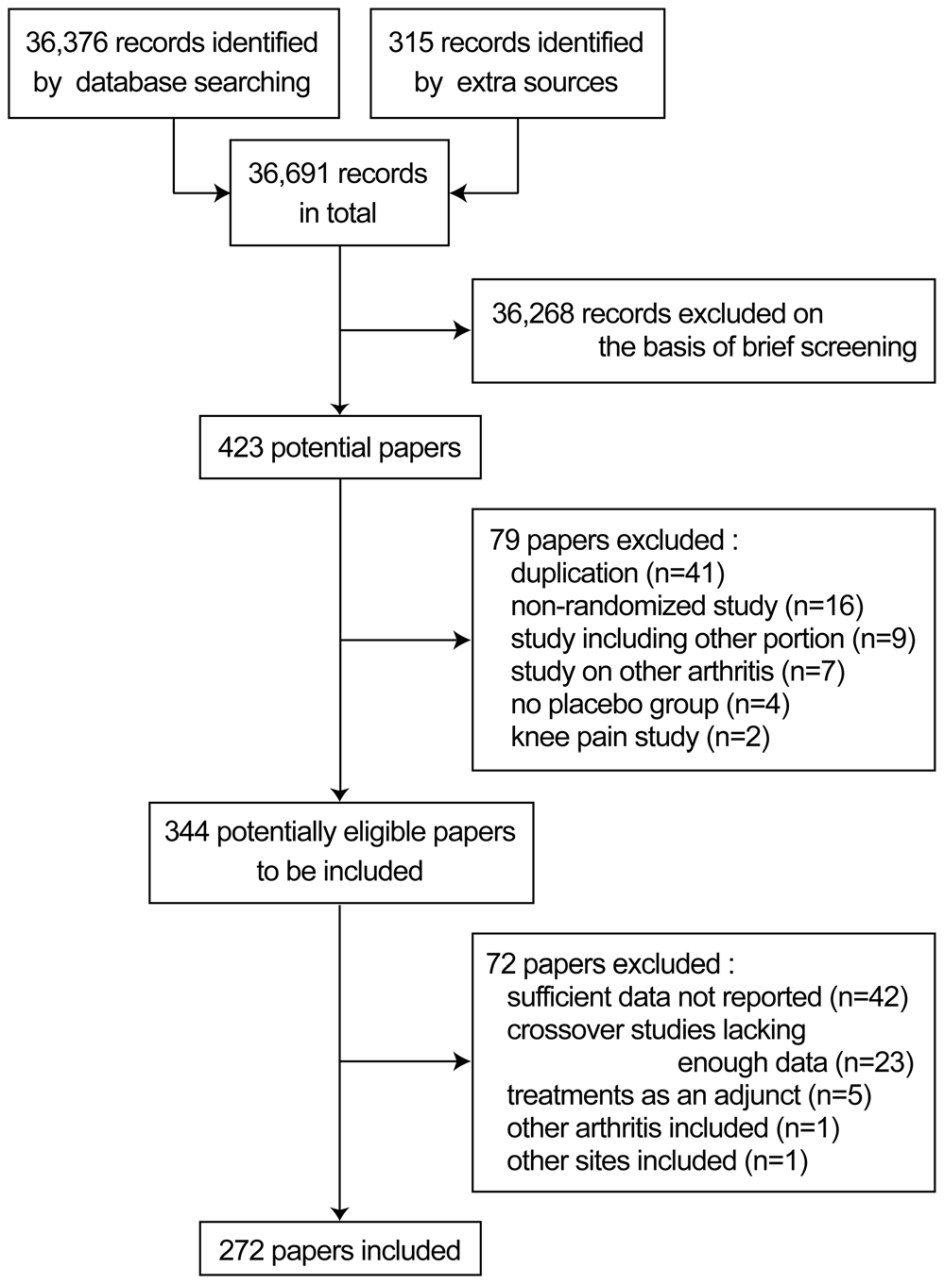
Study flow diagram.

We identified 36,691 records (PubMed, 1646; SCOPUS, 32,246; Cochrane Registered Trials, 2484; and other source, 315), of which 344 papers were identified as potentially eligible for our analyses (Figure 1). Of these, 72 papers were excluded because they lacked sufficient data (n = 42), were crossover studies not reporting enough data (n = 23), used test treatments as adjuncts (n = 5), included rheumatoid arthritis patients (n = 1), or reported data combined over the hip and knee joints (n = 1). Finally, a total of 272 papers reporting 281 trials were analyzed.

Information regarding the trials included in our analysis is provided in Table 1. Diverse treatment strategies such as pharmacological, non-pharmacological, and surgical therapies were used. While these treatments were commonly investigated in trials without a flare design, they were evenly tested in trials with and without prohibition of usual analgesics or in trials with and without allowance of escape medication. The median trial duration was 12 weeks (interquartile range: 6 to 24) and the median time for provision of final placebo was 6 weeks (interquartile range: 3 to 12). In total, 44,976 patients were considered for the active groups and 22,284 for the placebo groups.

**Table 1:** Characteristics of the included trials.

	No. of paper (No. of trial)
**Total study**	272 (281)
**Treatment type**	
Pharmacological	203 (212)
Non-pharmacological	68 (68)
mixed	1 (1)
**Delivery route** *	
Oral	127 (135)
Topical	64 (64)
Percutaneous	71 (72)
Surgical	4 (4)
**Flare design**	
Yes	26 (26)
No/unclear	246 (255)
**Prohibition of usual analgesics**	
Yes	144 (152)
No/unclear	128 (129)
**Allowance of escape medication**	
Yes	135 (139)
No/unclear	137 (142)

No., number.

*Six studies administered treatments using mixed methods.

### Primary analyses

**Figure 2:**
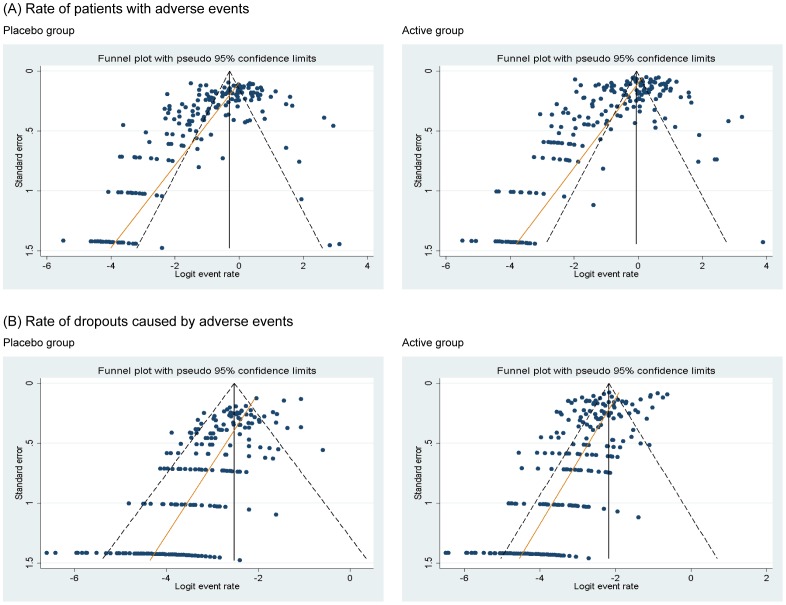
Funnel plots including an Egger's regression line.

The summary rates of patients with AEs were 31.8% (95% confidence interval: 29.1 to 34.7; I^2^ = 96%) in the active groups and 27.4% (24.7 to 30.3; I^2^ = 92%) in the placebo groups. The rate of the placebo groups accounted for 86.2% of the rate of the active groups. Using Egger's regression test, bias was detected in both groups (P<0.001 for each) (Figure 2). The summary rates of dropouts caused by AEs were 5.2% (4.6 to 5.9; I^2^ = 87%) in the active groups and 4.8% (4.3 to 5.4; I^2^ = 60%) in the placebo groups. The rate of the placebo groups accounted for 92.3% of the rate of the active groups. Using Egger's regression test, bias was found in both groups (P<0.001 for each) (Figure 2).

**Figure 3:**
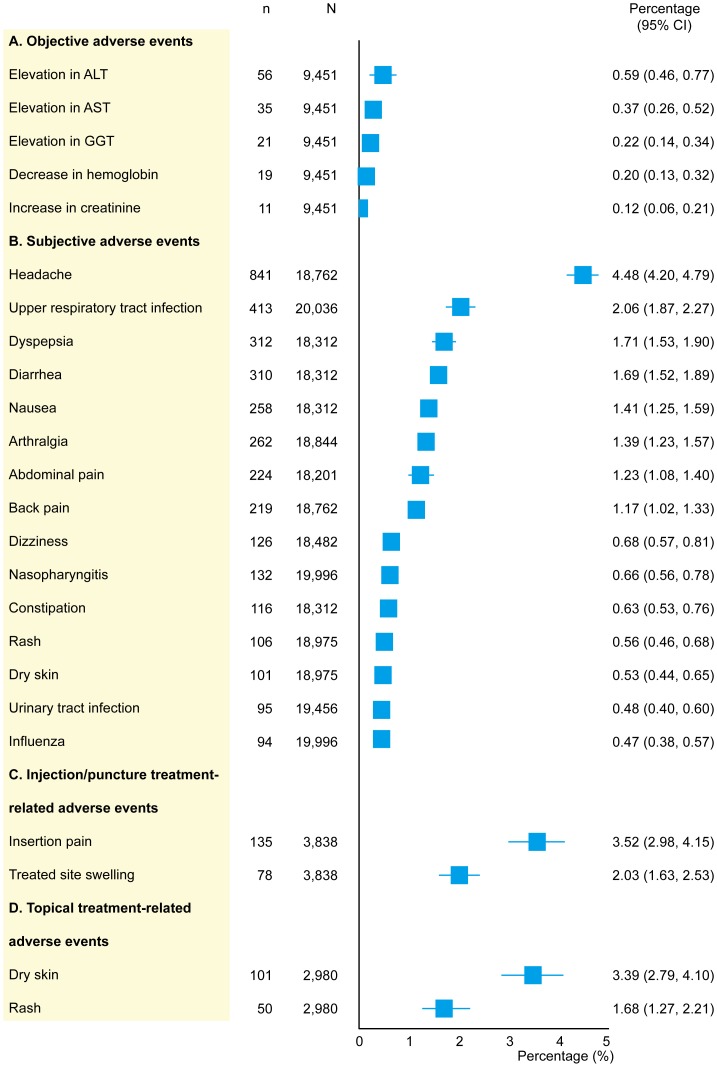
Rates of nonspecific adverse events. n, number of patients reporting adverse events; N, total sample size; ALT, alanine aminotransferase; AST, aspartate aminotransferase; GGT, gamma-glutamyltransferase.

The profiles of nonspecific AEs are shown in Figure 3. Abnormalities in hemoglobin, creatinine, and liver enzymes were commonly identified objective AEs. Elevated alanine aminotransferase was the most commonly reported AE, with a rate of 0.59% (0.46 to 0.77). With regard to subjective AEs, a variety of symptoms pertaining to different parts of the body were reported. Headache was the most frequent AE, with a rate of 4.48% (4.20 to 4.79). For injection/puncture treatment-related nonspecific AEs, insertion pain was the most commonly reported, with a rate of 3.52% (2.98 to 4.15), followed by treated site swelling. When topical treatment-related nonspecific AEs were examined, dry skin was the most common symptom, with a rate of 3.39% (2.70 to 4.19), followed by rash.

### Secondary analyses

When the analysis was separately restricted to studies with clearly concealed allocation (n = 109), explicit blinding of patients (n = 249) or providers (n = 73), or adequate dropout rate (n = 135), the significance of the rates of patients with AEs and dropouts caused by AEs remained unchanged. Furthermore, when the analysis was performed across trials that met all factors (n = 28), the respective rates were 24.9% (17.0 to 34.9; I^2^ = 85%) and 3.9% (2.8 to 5.3; I^2^ = 0%).

**Figure 4:**
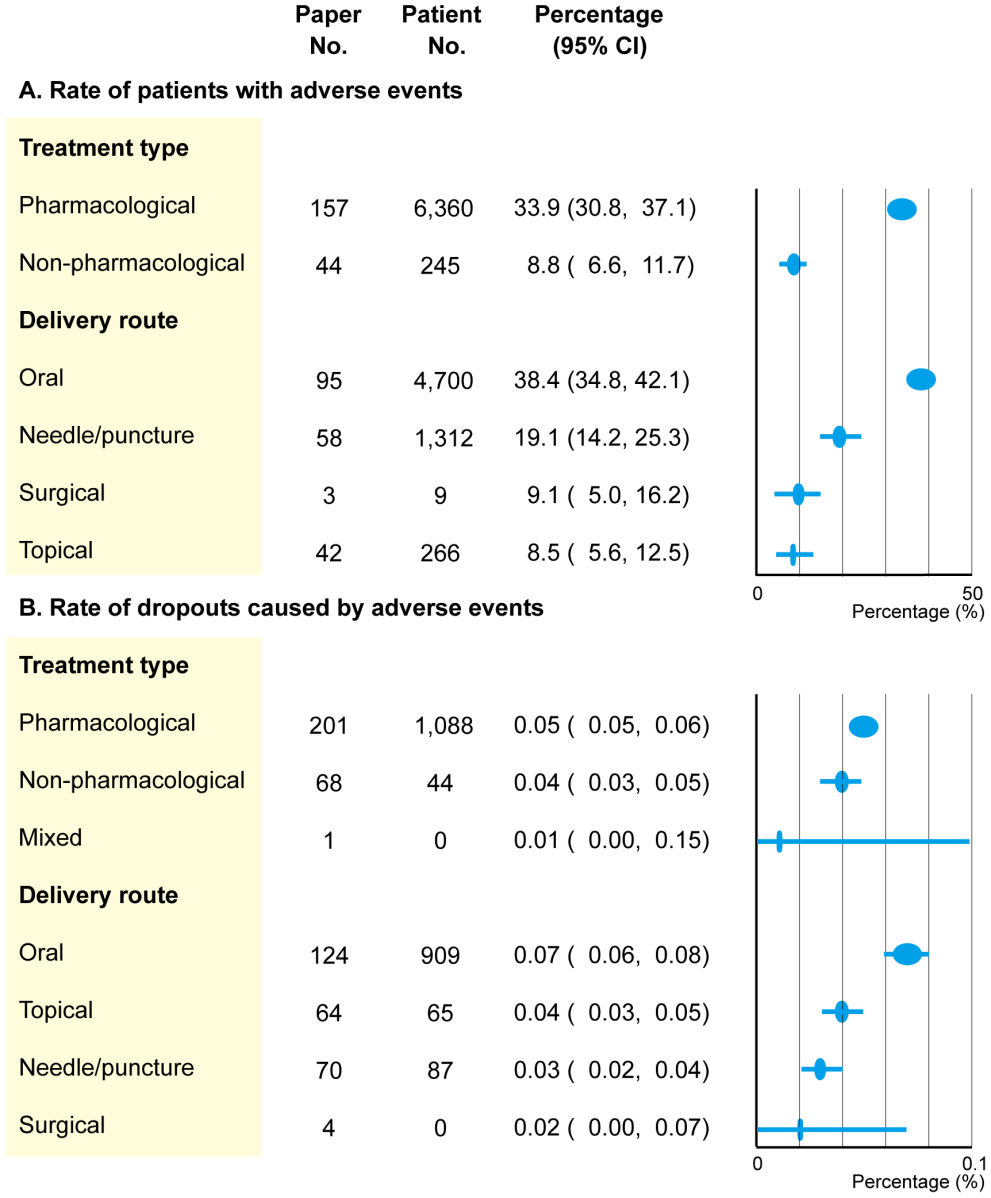
Rates for treatment type and delivery route.

When the rate of patients with AEs was categorized by the treatment type, a linear trend was observed (P<0.001) (Figure 4). Pharmacological treatment had the largest impact on AEs, followed by non-pharmacological treatment. The rate decreased consistently for delivery route (P<0.001), with the highest number of AEs experienced for oral delivery, followed by injection/puncture, surgical, and topical routes.

When the rate of dropouts caused by AEs was categorized by the treatment type and delivery route, a linear trend was observed for both factors (Figure 4). The rate decreased (P = 0.017) in pharmacologic treatment studies, followed by non-pharmacological and mixed treatment studies. Likewise, a consistent decrease was observed (P<0.001) when the delivery routes were considered in the following order: oral, topical, injection/puncture, and surgical.

The associations of two variables with the trial duration and study design are summarized in Table 2. The rate of patients with AEs was significantly associated with the trial duration (P = 0.021), flare design (P = 0.027), prohibition of usual analgesics (P = 0.003), and allowance of escape medication (P<0.001). In contrast, the rate of dropouts caused by AEs was only associated with the trial duration (P = 0.004).

**Table 2:** Results of the meta-regression analysis.

	Rate of patients with adverse events	Rate of dropouts caused by adverse events
	Coefficient (95% CI)	Coefficient (95% CI)
	P value	P value
**Continuous outcome**		
Trial duration (per week)	0.013 (0.002, 0.023)	0.006 (0.002, 0.010)
	P = 0.021	P = 0.004
**Binary outcome** *		
Flare design	0.784 (0.088, 1.480)	0.182 (−0.156, 0.521)
	P = 0.027	P = 0.291
Prohibition of usual analgesics	0.694 (0.240, 1.148)	0.038 (−0.208, 0.283)
	P = 0.003	P = 0.764
Allowance of escape medication	0.935 (0.500, 1.370)	0.003 (−0.238, 0.245)
	P<0.001	P = 0.979

CI, confidence interval.

The coefficients are values after logit transformation.

*For binary outcomes, “no/unclear” and “yes” were coded as 1 and 2, respectively.

## Discussion

Previously, numerous reviews have reported that the rate of patients with AEs and the rate of dropouts caused by AEs in placebo groups were 10–50% and 2–6%, respectively [5]–[15], [36]. When these rates were compared with those in the active groups, the rate of patients with AEs in the placebo groups was 72.8–81.7% of that in the active groups [8]–[12]. With respect to the rate of dropouts caused by AEs, the rate in the placebo groups was 43.9–99.6% of that in the active groups [6], [11]. Our findings of 86.2% and 92.3% correspond with these other studies. All of these data imply that the nonspecific AEs may substantially account for the development of AEs in the active groups.

Previous reviews have also investigated the profiles of nonspecific AEs caused by the nocebo response [5]–[15]. According to these reviews, subjective nonspecific AEs included a number of conditions occurring throughout the body, and numerous events were significant. Our study also confirms these findings. Meanwhile, to our knowledge, none of the previous reviews investigated objective AEs. Our study found that the rates of some objective AEs were significant. Considering that these nonspecific AEs occurred in patients receiving inert placebos, the findings are surprising. Although these results may be explained by many factors (e.g., participation burden), previous studies have focused on the nocebo response linked to expectation and conditioning theories.

First, some reviews found that nonspecific AEs reflected the pattern of AEs caused by active treatments, suggesting that the nonspecific AEs may be triggered by expectation [5], [7], [8]. Expectation of AEs caused by active treatments may generate similar AEs in patients receiving placebos. To check whether the patterns of AEs were similar between the two groups in our study, we conducted correlation analyses with regard to the rates of patients with AEs, rates of dropouts caused by AEs, and rates of patients for some AEs. We found significant relationships for all factors (Table S1 and figure S1), which corresponds with the previous findings [5], [7], [8].

Second, some reviews indicated the possibility of conditioning-mediated nonspecific AEs [8]. Nonspecific AEs may occur in patients who were previously exposed to similar treatments and thus experienced AEs. There is evidence that, when unconscious physiological functions such as hormonal secretion are involved, nocebo responses are mediated by conditioning [37]. However, verbally-induced expectations have no effect on unconscious physiological functions [37]. Therefore, our finding of significant objective nonspecific AEs may be explained by the conditioning theory, although we could not analyze how many patients were previously conditioned.

In our study, the magnitude of the nocebo response was found to be affected by the treatment type, delivery route, and study design. Specifically, the rate of patients with adverse events was closely related to the study design. Some trials described that, although escape medications were allowed for treating knee flare pain during trials, they were instead used to treat AEs [38], [39]. This suggests that the nonspecific AEs may have been affected by strategies to deal with patients complaining of pain during the trials. We addressed this point, and found that a higher rate of patients with AEs was associated with longer trial duration, flare design, prohibition of usual analgesics, and allowance of escape medication. Hence, we think that escape medication may act as an alert, even when adverse events were minimal and could be ignored.

Although our study provides an excellent opportunity to study nonspecific AEs, the results should be interpreted with caution. First, we encountered different reporting styles across trials. Some trials enumerated AEs reported by >2% of patients in any group, and others by >5% of patients. In some cases, only generic AEs (e.g., gastrointestinal symptoms) were presented. We excluded data pertaining to generic AEs for practical reasons, while the remaining data were encoded as reported. Therefore, if all AEs were reported in trials, the rates of nonspecific AEs would have been greater.

Second, the data collection methods for subjective AEs were insufficiently detailed in trials. According to a study [40], patients who reported no side effects in an open questionnaire reported at least one side effect in a structured questionnaire. This suggests that the data collection methods can influence the rates of AEs. However, most of the trials in our study either did not report the data collection methods or did not use a structured checklist. As our results may be confounded by these factors, it is necessary to clearly report the data collection methods in future trials.

The final major limitation includes the small study effects, in which smaller studies in a meta-analysis show larger treatment effects. Although the impact of such biases has been well demonstrated for efficacy outcomes [17], it has not yet been discussed for safety outcomes. Nonetheless, it is possible that smaller studies with greater AE rates may have a rare chance to be published in journals. For this reason, it is possible that our results may have been affected.

We believe that the present findings will have several useful implications for the design and conduct of clinical trials. First, substantial efforts have recently been directed toward reducing the magnitude of the nocebo response at the patient level, such as excluding patients with a higher risk of experiencing the nocebo response [36], [41], and carefully explaining the possible AEs during a trial [36], [42], [43]. However, our findings imply that adopting an optimal study design (i.e., short trial duration, non-flare design, allowance of usual analgesics, and prohibition of escape medication) may be another option for reducing the nocebo response without intervening in patient-researcher interactions.

Second, in most trials, the patient recruitment strategies were based on a power calculation that aimed to demonstrate the efficacy of active treatments. Nonetheless, conclusions regarding the safety outcomes were presented on the basis of those sample sizes. However, such sample sizes may be insufficient to demonstrate the safety of active treatments, thereby leading to a type II error. For example, the continuous form of the odds ratio for patients with or without AEs between the two groups was 0.1 in our study. When compared with the effect sizes for the effectiveness of all treatments presented in a guideline study [3], this magnitude is very small. Therefore, caution should be observed when interpreting the safety outcomes in clinical trials.

Similarly, it can also be argued that most of the active treatments are safe, because most AEs seen in the treatment groups can be considered to be nonspecific. However, one study demonstrated that some AEs were frequently reported in the German population not taking any drugs [44]. Specifically, headache, back pain, and joint pain were experienced by >10% of the general population. When these data are compared with our data, the rates of AEs in our study are lower than those in the German population. As general clinical trials may report lower rates for AEs [6], [8], the safety of active treatments should be determined on the basis of fully-powered clinical trials.

## Conclusions

From the data based on 281 knee osteoarthritis trials, we found that nonspecific AEs substantially accounted for the development of AEs in the active groups. They accounted for 86.2% of the active groups for the rate of patients with AEs and 92.3% of the active groups for the rate of dropouts caused by AEs. In addition, nonspecific AEs included conditions involving the entire body, including musculoskeletal, gastrointestinal, respiratory, urogenital, cerebrovascular, psychiatric, and immune system manifestations. Upon further investigation, the rate of patients with AEs was associated with the treatment type, delivery route, and study design. We believe that our findings provide some clues toward reducing nonspecific AEs in the design and conduct of clinical trials.

## Supporting Information

Figure S1 **Plots of patient rates in the treatment groups against patient rates in the placebo groups.**
(TIF)

Table S1 **Correlation coefficients.**
(DOCX)

Checklist S1 **PRISMA checklist.**
(DOC)
