# Supplementary figures and images for: Nonspecific Adverse Events in Knee Osteoarthritis Clinical Trials: A Systematic Review
Source: PLoS One. 2014 Nov 3;9(11):e111776. doi: 10.1371/journal.pone.0111776 (PMC4218813; doi:10.1371/journal.pone.0111776)

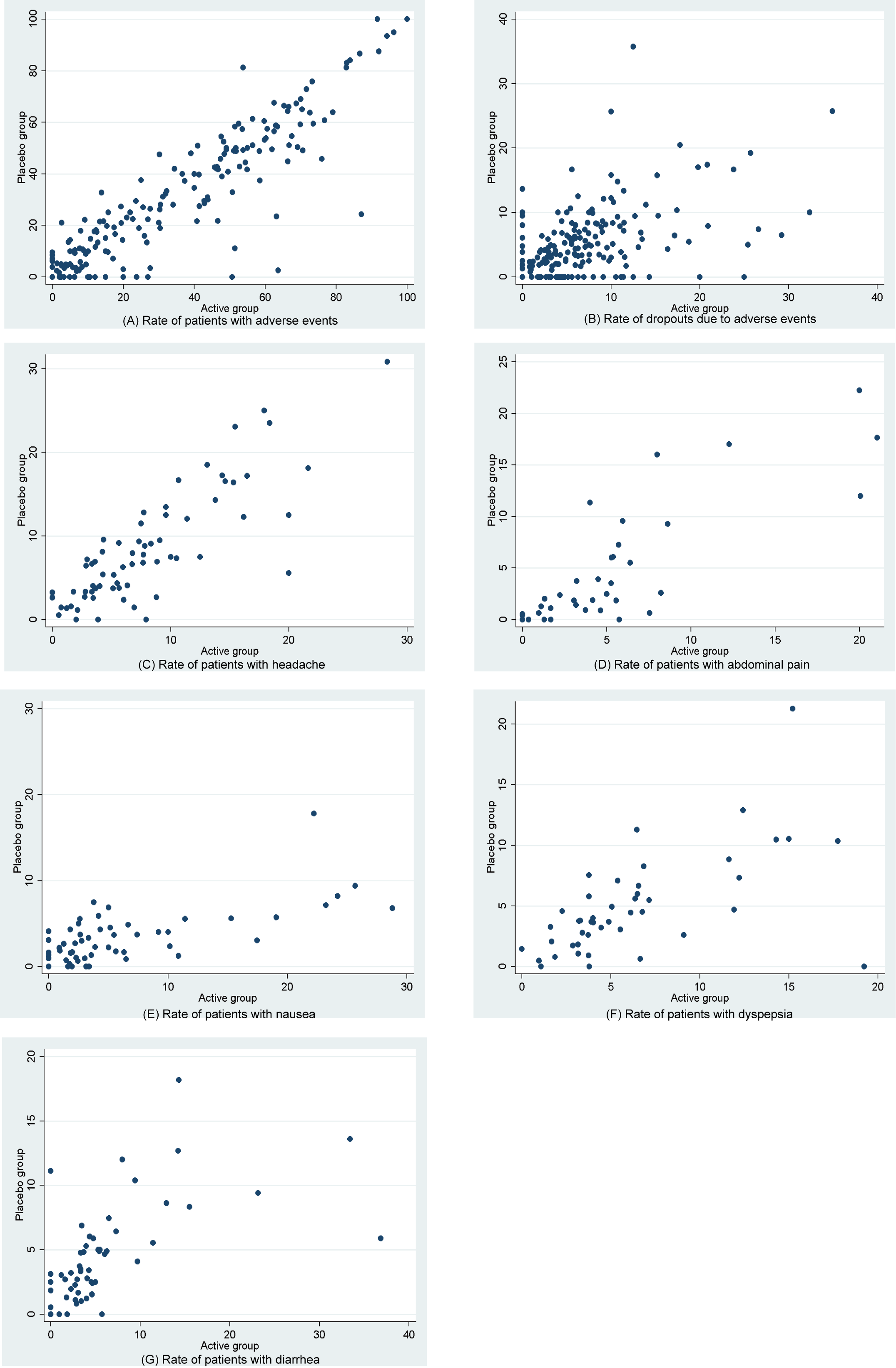

Supplement: Figure S1 — Plots of patient rates in the treatment groups against patient rates in the placebo groups. (TIF) [file pone.0111776.s001.tif]
