# Supplementary material for: Nonspecific Adverse Events in Knee Osteoarthritis Clinical Trials: A Systematic Review
Source: PLoS One. 2014 Nov 3;9(11):e111776. doi: 10.1371/journal.pone.0111776 (PMC4218813; doi:10.1371/journal.pone.0111776)
Supplement: Table S1 — Correlation coefficients. (DOCX) [file pone.0111776.s002.docx]

Supplementary table S1. Correlation coefficients

|  | Study number | Coefficient | P value |
| --- | --- | --- | --- |
| Rate of patients with adverse events | 269 | 0.908 | P < 0.001 |
| Rate of dropouts caused by adverse events | 204 | 0.604 | P < 0.001 |
| Rate of patients with headache | 68 | 0.827 | P < 0.001 |
| Rate of patients with abdominal pain | 37 | 0.825 | P < 0.001 |
| Rate of patients with nausea | 58 | 0.671 | P < 0.001 |
| Rate of patients with dyspepsia | 45 | 0.602 | P < 0.001 |
| Rate of patients with diarrhea | 55 | 0.586 | P < 0.001 |
